# A block mixture model to map eQTLs for gene clustering and networking

**DOI:** 10.1038/srep21193

**Published:** 2016-02-19

**Authors:** Ningtao Wang, Kirk Gosik, Runze Li, Bruce Lindsay, Rongling Wu

**Affiliations:** 1 Department of Biostatistics, University of Texas School of Public Health, Houston, TX 77030, USA; 2 Department of Public Health Sciences, The Pennsylvania State University, Hershey, PA 17033, USA; 3 Department of Statistics, The Pennsylvania State University, University Park, PA 16802, USA

## Abstract

To study how genes function in a cellular and physiological process, a general procedure is to classify gene expression profiles into categories based on their similarity and reconstruct a regulatory network for functional elements. However, this procedure has not been implemented with the genetic mechanisms that underlie the organization of gene clusters and networks, despite much effort made to map expression quantitative trait loci (eQTLs) that affect the expression of individual genes. Here we address this issue by developing a computational approach that integrates gene clustering and network reconstruction with genetic mapping into a unifying framework. The approach can not only identify specific eQTLs that control how genes are clustered and organized toward biological functions, but also enable the investigation of the biological mechanisms that individual eQTLs perturb in a signaling pathway. We applied the new approach to characterize the effects of eQTLs on the structure and organization of gene clusters in *Caenorhabditis elegans*. This study provides the first characterization, to our knowledge, of the effects of genetic variants on the regulatory network of gene expression. The approach developed can also facilitate the genetic dissection of other dynamic processes, including development, physiology and disease progression in any organisms.

An essential step toward constructing the genotype-phenotype map is to understand how DNA polymorphisms affect variation in a phenotype by perturbing transcripts, metabolites and proteins^1^. There has been an avalanche of genomic studies that characterize gene expression and its roles in linking genetic variants to phenotypic formation^2^,^3^,^4^,^5^,^6^. The functional consequences of gene transcripts are usually studied by first clustering their expression data into categories on the basis of functional similarity. Increasing research has been focused on understanding how changes in gene expression are encoded by expression quantitative trait loci (eQTLs) that are involved in particular biological processes^7^,^8^,^9^. Although many approaches have been developed for gene clustering and eQTL mapping, an integrative framework by which to chart a clear picture of genetic mechanisms regulating the function of gene expression has not been constructed. Such integration will not only facilitate our mechanistic understanding of differentiated gene expression in response to environmental clues, but also potentially increase the statistical power of genetic dissection of gene expression.

Here we established a unifying framework for simultaneously mapping eQTLs and clustering gene expression profiles in a segregating mapping population. A typical eQTL mapping data is composed of an *m*-dimension of gene transcripts for a set of individuals (*n*) genotyped by a *p*-dimension of DNA markers. The central idea of our framework is to class the *m*** × ***n* matrix into different latent blocks by each eQTL located within a pair of flanking markers. In computer science, a so-called block mixture model has been proposed to address such a row-column independent hierarchical clustering problem^10^,^11^,^12^. Based on the block mixture model, Kuruppumullage^13^ formulated a composite likelihood to obtain statistical specification and estimation of latent variables and blocks. We integrated unsupervised gene expression pattern discovery and interval mapping within the composite likelihood framework and further implemented the two-layer EM algorithm to localize the genomic locations of eQTLs and estimate their genetic effects on gene clustering and function. We proved the consistency of the latent class assignment function when the number of observations tends to infinity. The model was validated through simulation studies and by new discoveries from reanalyzing genetic and genomic data collected in a mapping population of *Caenorhabditis elegans*^5^.

The new model classifies different genes into distinct clusters and estimates expression amounts of each cluster for different genotypes at an eQTL detected. Therefore, the model can characterize how individuals eQTLs regulate the pattern of gene differentiation. By reconstructing the regulatory networks among gene clusters, the model provides results that facilitate our mechanistic understanding of how gene expression is mediated in response to developmental and environmental clues.

## Results

We used the new model to reanalyze a real example for genetic mapping of gene expression in *Caenorhabditis elegans* by Kruglyak’s group^5^. In a mapping population of 208 recombinant inbred advanced intercross lines from a cross between a laboratory strain (N2) and a wild isolate (CB4856) from Hawaii, 1455 SNP markers were genotyped and 20,401 transcripts measured. The microarray data, preprocessed through a normexp background correction, were normalized using quantile standardization. The transcripts with sample standard deviations < 0.9 were removed, after which we obtained a total of 5,450 transcripts for further modeling and analysis.

### Optimal number of gene clusters

**Figure 1:**
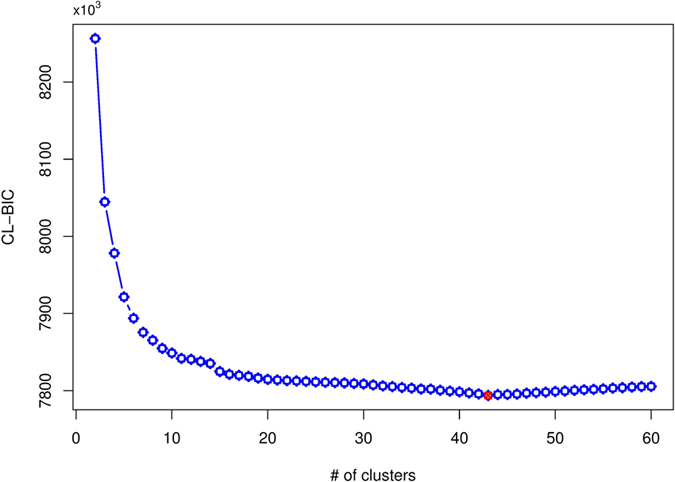
Plot of composite likelihood BIC values over the number of gene clusters identified by the block mixture model.

A mixture model has been used for gene clustering. A traditional approach is to classify all genes on a single genotype into different clusters based on their similarity. To cluster genes on multiple genotypes, expressed as a 208** × **5450 data matrix in our example, a Gaussian block mixture model is implemented and solved, with composite likelihood BIC as a model-selection criterion. In order to obtain the global maxima, multiple initial values were selected and compared. To the end, we obtained the composite likelihood BIC values under different numbers of gene clusters, from which the optimal number of gene clusters was found to be 43 (Fig. 1). The estimated optimal cluster number from the block mixture model is a consequence of the interactions between gene expression differences and genotypic differences.

### Detecting eQTLs for gene clustering

**Figure 2:**
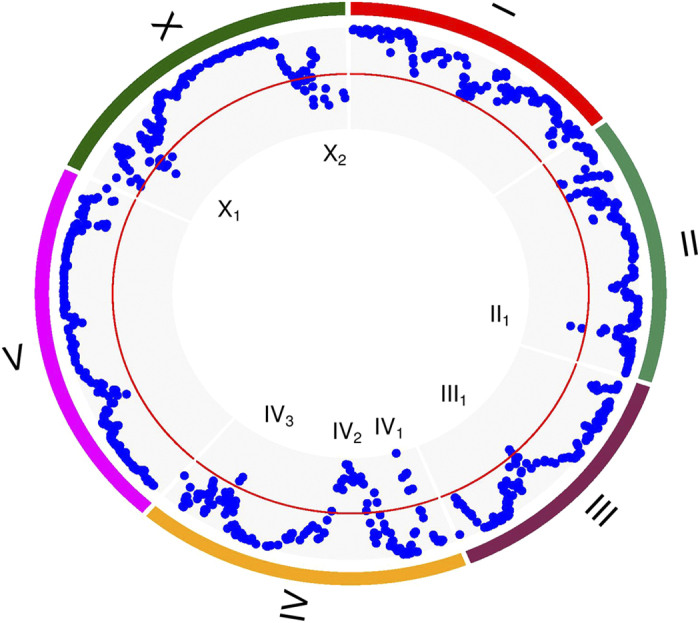
The genome-wide identification of significant eQTLs over six chromosomes (outer circle) in *C. elegans* by the block mixture model. The red line (inner circle) is the genome-wide critical threshold at the 5% significance level determined from permutation test. Significant eQTL spots, denoted by Roman letters with subscripts, were detected in chromosome II, III, IV, and X.

The effect of an eQTL on gene clustering can be characterized by testing whether any one of the clusters has different mean values of gene expression between two genotypes at this eQTL bracketed by a pair of flanking SNPs. If such a difference is significant, then we claim that this eQTL is significantly associated with gene clustering. We implemented this testing procedure to scan all SNPs across six chromosomes of the *C. elegans* genome, obtaining a total of 52 clustering-related eQTLs, with 2, 2, 27, and 21 distributed on chromosome II, III, IV, and X, respectively (Fig. 2). Chromosome IV has three distinct eQTL spots, labeled as IV_1_, IV_2_, and IV_3_, of which IV_2_ and IV_3_ were also observed by a simple single marker/single gene association analysis in Rochman *et al.*’s (2010) original study whereas IV_1_ and IV_2_ observed by Chun and Keles’s multivariate sparse partial least squares regression (M-SPLS) regression^14^. Of the two eQTL spots detected on sex chromosome X from our approach, one, denoted as X_1_, was detected by the simple association analysis and the other, denoted as X_2_, detected by M-SPLS regression. The results from our approach cover different results detected by the two existing ones, respectively, suggesting that our approach is more general for eQTL mapping. A spot in chromosome II, labeled as II_1_, and a spot in chromosome III, labeled as III_1_, were detected only by our approaches, demonstrating its statistical power.

### Pleiotropic eQTLs and eQTL × cluster interactions

**Figure 3:**
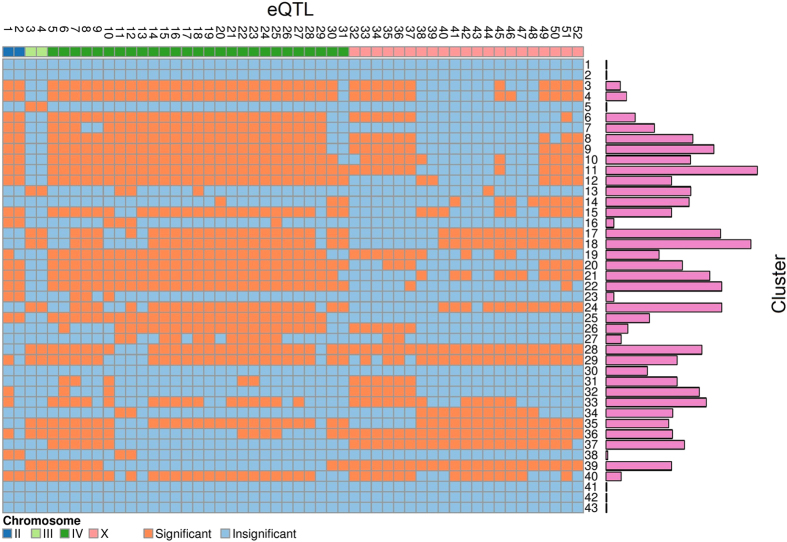
Distribution of significant genetic effects on 43 gene clusters by 52 eQTLs located on chromosome II, III, IV, and X in *C. elegans*, combined with gene cluster sizes.

An eQTL may be only responsible for a particular set of gene clusters. Through hypothesis testing (*ii*), we obtained the number and type of gene clusters that are controlled by each eQTL detected (Fig. 3). If an eQTL simultaneously affects more than one cluster, this eQTL is thought to be pleiotropic. The number of gene clusters affected by an eQTL is used to define the pleiotropic capacity of this eQTL. Spot IV_1_ and IV_2_ contains many strongest pleiotropic eQTLs, affecting the largest number of clusters (15–28), followed by those in II_1_ (18–23), X_1_ (16–20), IV_3_ (14–18), X_2_ (9–20) and III_1_ (11) in order. Different eQTL spots may affect the same gene clusters, but with a large variation; for example two different spots may affect the same clusters as many as 28 or as few as 0. These similarities and differences of pleiotropic control by eQTLs are related to the genetic machineries of developmental modularity.

**Figure 4:**
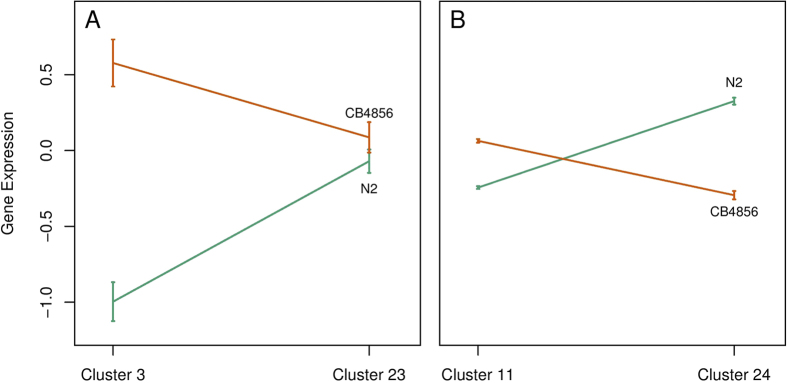
Significant genotype by cluster interactions at eQTL IV_2_ 6260291 expressed as difference in the magnitude (**A**) of genetic effect or the direction (**B**) of genetic effect on different gene clusters. At an eQTL, there are two homozygous genotypes each with the two same alleles inherited from a parent, a laboratory strain (N2) or a wild isolate (CB4856).

It can be seen from Fig. 3 that a single cluster may be controlled by multiple eQTLs although some involves more eQTLs than others. Cluster 28 is controlled by the largest number of eQTLs, whereas cluster 30 includes the smallest number. Most pairs of clusters share the same eQTLs as the common genetic basis for the correlations among these cluster pairs. An eQTL may pleiotropically affect two different clusters, but the magnitude of its effect may vary from one cluster to other, leading to significant eQTL** × **cluster interactions. From hypothesis test (*iii*), we found that all significant eQTLs detected display pronounced interactions between genotypes and clusters. An eQTL may interact with clusters by changing the magnitude or direction of gene expression values between different clusters. Also, the same eQTL may exert genotype** × **cluster interactions with different patterns over different cluster pairs. For example, eQTL IV_2_ 6260291 displays genotype** × **cluster interactions through difference in the magnitude of its genetic effect on cluster 3 and 23 but in the direction of its genetic effect on cluster 11 and 24 (Fig. 4).

**Figure 5:**
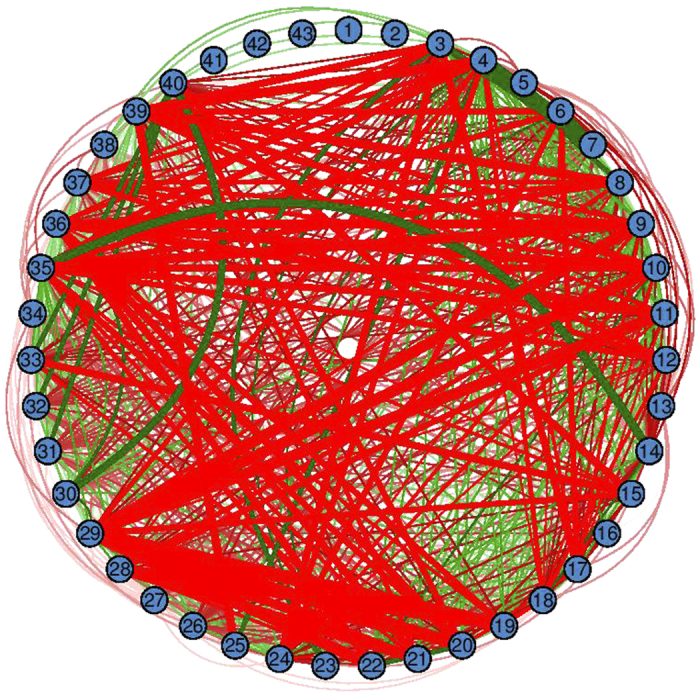
Ubiquitous occurrence of eQTL by cluster interactions over all possible pairs of clusters. Green and red lines denote genotype by cluster interactions due to difference in the magnitude and direction of genetic effects, respectively, on a particular cluster pair. The thickness of the lines are proportional to the frequency of genotype by cluster interactions at 52 eQTLs.

Direction-varying interactions are more ubiquitous and stronger than magnitude-varying interactions. Figure 5 illustrates the numbers of eQTLs that display magnitude- and direction-varying genotype** × **cluster interactions over cluster pairs. Direction-varying interactions pervade cluster pairs, showing a considerable amount of genotypic variation in the differentiated expression of different clusters related to particular biological functions.

### eQTLs for cluster structure and network

**Figure 6:**
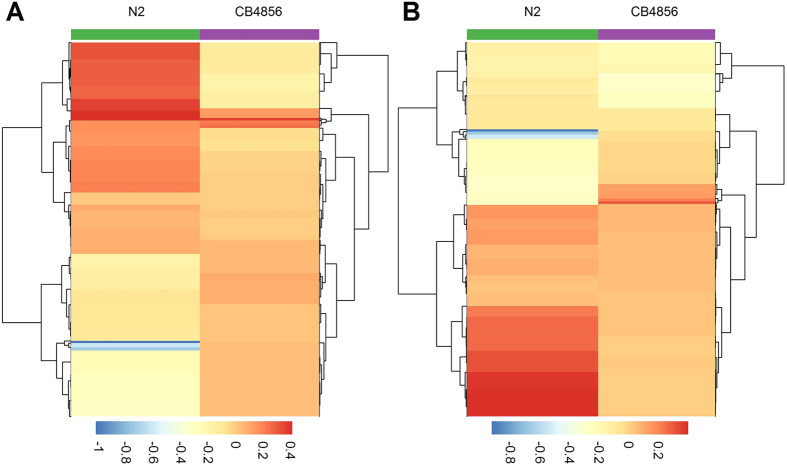
Heatmaps of 43 gene clusters who co-expression pattern varies depending on the genotype at an eQTL. Examples are derived from VI_1_ 6461993 (**A**) and X_1_ 16327274 (**B**), at each of which two homozygous genotypes each with the two same alleles were inherited from a parent, a laboratory strain (N2) or a wild isolate (CB4856).

We obtained 43 distinct clusters, but these clusters may have complex mutual relationships. Our approach allows us to test how an eQTL controls the structure of relationships among the clusters. Through hypothesis test (*iv*), we elucidate the difference in cluster relationships between different genotypes at a particular eQTL detected. Figure 6 shows two examples in which two eQTLs from spot VI_1_ and X_1_ affect the structure of clusters. Some clusters are close to each other in one genotype, but they tend to be far away in the alternative genotype at the same eQTL.

**Figure 7:**
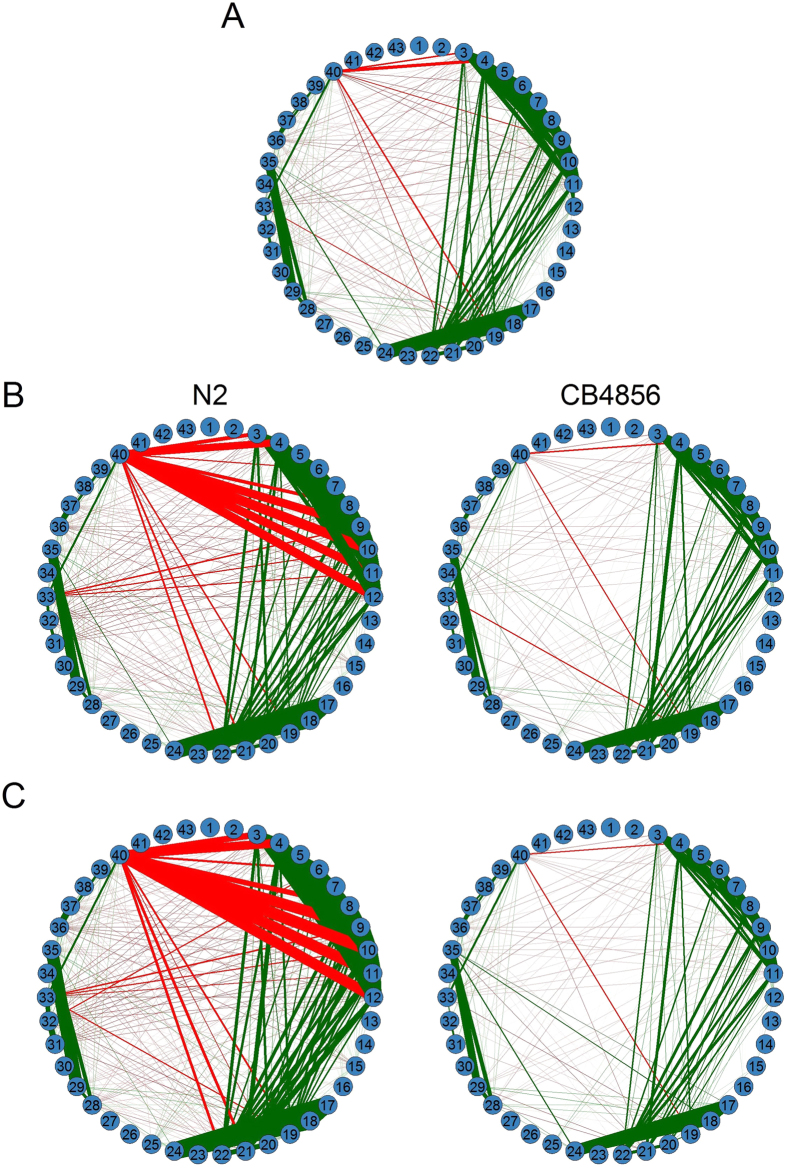
Regulatory network of 43 gene clusters for the overall mapping population (**A**), but with structure and organization affected by eQTLs, e.g., VI_1_ 6461993 (**B**) and X_1_ 16327274 (**C**), at each of which two homozygous genotypes each with the two same alleles were inherited from a parent, a laboratory strain (N2) or a wild isolate (CB4856). Green and red lines denote positive and negative correlations between two particular clusters, respectively, with the thickness of lines associated with the degree of correlation.

The mutual relationships among all clusters can be expressed in terms of co-expression networks. Overall, 43 clusters form a complex web of mutual relationships, with some clusters being more closely related with each other than with others (Fig. 7A). The fundamental structure and organization of this web are controlled by eQTLs. For example, the genotype of eQTL VI_1_ 6461993 inherited from parent CB4856 (a wild isolate) is much more sparse in network structure, compared with the alternative genotype from parent N2 (a laboratory strain) (Fig. 7B). In the genotype composed of the CB4856 alleles, cluster 40 only displays a few weak connections with other clusters, but this cluster connects with many more other clusters much more strongly. Similar discrepancies can be seen for other clusters. In a second example, where the two genotypes at eQTL X_1_ 16327274 are compared, the mutual connectivity of all clusters within the network exhibits strong genotype-dependent differences (Fig. 7C), showing the impact of this eQTL on the structure of gene expression network.

### Result validation by computer simulation

We performed simulation studies to demonstrate the satstiatical properties of our newly developed block mixture model. We simulated a backcross mapping population of *n* = 208 progeny by mimicking the example used above. For all these progeny, the classification of simulated normally distributed 5,450 transcripts into 43 clusters was assumed to be controlled by an eQTL located at 24 cM from the left end of a linkage group. The linkage group is 50 cM long, composed of six evenly spaced markers. The position at which the largest composite likelihood ratio test statistic is obtained is viewed as the estimate of the eQTL position. The simulation and estimation were performed 500 replicates, obtaining the maximum likelihood estimates of all parameters and their sampling errors. The results suggest that the number of gene cluster can be correctly estimated; among 500 simulation replicates 480 (96%) obtained the correct estimate of gene cluster number (Supplementary Table S1). The estimates of all model parameters are reasonably unbiased and precise. The proportions of each gene cluster can be well estimated, even for those with a low proportion. The position of the eQTL was estimated as 23.70 ± 1.17, in an agreement with true position 24. Genotypic values of each gene cluster were reasonably estimated, showing that the block mixture model is powerful for the estimation of genetic effects by an eQTL.

**Figure 8:**
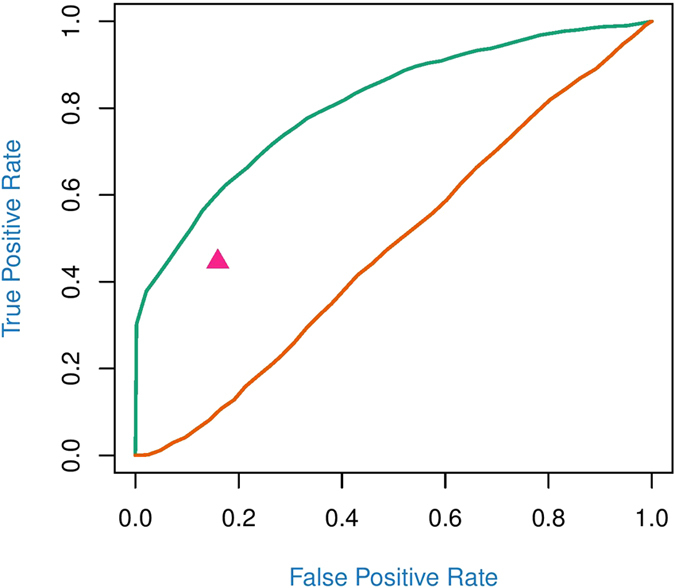
ROC curves for hotspots detection using block mixture model (green line), MOM^15^ (brown line), and M-SPLS^14^ (purple star) from 500 simulated replicates. For M-SPLS, type I error and power were calculated conditionally on the penalized latent vector components.

We compared the performance of the new model with those of existing approaches, mixture over marker^15^ and multivariate sparse partial least squares regression^14^, through additional simulation. The new model shows much higher power of detecting a significant eQTL than existing approaches (Fig. 8).

## Discussion

To better respond to environmental perturbations, the organism would modify their developmental and physiological programs through regulating and coordinating the pattern of its gene expression across various cell and tissue types^6^,^16^. Because of this underlying mechanism, analysis and modeling of differentiated expression profiles of genes have been widely used as an essential procedure to unravel the biological organization and function of living organisms. As one of the first steps in gene expression analysis, clustering is aimed to classify genes into different clusters on the basis of their similarity and dissimilarity in terms of biological function^17^. Because different genes are often co-expressed in facing environmental stimuli, an increasing body of studies has explored the reconstruction of transcriptional gene regulatory networks in which causal relationships of different genes can be elucidated^18^,^19^. The pattern of how genes are expressed singly or jointly with other genes in a complex manner to achieve biological functions is attributed to polymorphic regions of the genome, which are known as expression quantitative trait loci (eQTLs). The identification of eQTLs, as one of the hottest topics in current genomic research, has been instrumental in shedding light on the genetic mechanisms underlying various biological and biochemical processes^6^,^20^,^21^,^22^,^23^.

Despite these developments of gene expression analysis, however, there has been no study that can integrate the strengths of gene clustering, network construction and eQTL mapping into an organizing framework. In this study, we have developed this framework by implementing a block mixture within a genetic mapping setting. While traditional eQTL mapping can identify specific eQTLs responsible for the expression of individual genes^5^, the new model can characterize how eQTLs regulate the similarity and dissimilarity of gene expression and the causal network of different genes. This new model displays a tremendous methodological breakthrough embodied in the two following aspects: First, existing gene clustering approaches classify tens of thousands of genes recorded on a single biological entity, such as a cell type, an organ, a treatment, or an individual. Although considerable efforts have been made to cluster genes simultaneously on several entities^24^,^25^,^26^, no studies thus far have been able to tackle gene clusters on a high-dimensional set of entities which contain unknown latent components. The new model capitalizes on the advantage of a block mixture model for the simultaneous identification of latent gene clusters or latent genotypes from high-dimensional genes** × **high-dimensional genotype expression data. Second, by considering all possible combinations, existing eQTL mapping is based on a single marker/single gene association analysis, which neglects the complex correlations of different genes. Our model regresses tens of thousands of genes on marker genotypes by virtue of a clustering procedure, allowing the differences of gene expression levels for each cluster to be compared and tested between different genotypes at an eQTL.

After significant eQTLs were found by the block mixture model, we have formulated a detailed procedure of testing how a specific eQTL controls individual gene clusters and their causal relationships. This procedure includes several hypothesis tests of fundamental biological relevance. For example, how a single eQTL pleiotropically affects multiple gene clusters can be tested; by testing all eQTLs a pleiotropic network of genetic control can be charted, greatly enhancing our understanding of the genetic architecture of gene expression. For a gene cluster of particular biological function, how many eQTLs are involved can be tested. This allows us to illustrate a polygenic picture of gene expression. We can also test whether there exist significant eQTL** × **gene cluster interactions. The same eQTL may affect two gene clusters differently through altering the magnitude or direction of expression levels from one gene to another. In sum, the new model, beyond existing clustering and mapping approaches, provides an unprecedented opportunity to understand the genetic variation of gene expression in depth.

We applied the new model to reanalyze the genetic data of gene expression collected by Rockman *et al.*^5^ in a mapping population of 208 recombinant inbred advanced intercross lines derived from a controlled cross of two *C. elegans* strains. In the original study, the authors identified thousands of significant eQTLs by using a regression analysis of single transcripts on single genes. The block mixture model classified 5,450 transcripts into 43 distinct clusters jointly based on 208 lines and identified 52 significant QTLs that determine the pattern of gene clustering. Each cluster can be regarded as a developmental module composed of many genes of similar function. It was found that each module is controlled by many eQTLs, and each eQTL pleiotropically affects multiple modules, but with the magnitude and direction of pleiotropic effects depending on the type of module (Fig. 3). eQTLs were found to regulate the structure and organization of networks constructed by gene clusters, suggesting their critical role in mediating the regulatory mechanisms of cellular and biological processes. To validate the biological relevance of the discoveries by the block mixture model, we performed computer simulation by mimicking the data structure of the mapping study for *C. elegans*. The model has adequate power for eQTL identification and displays low false positive rates.

The model can be extended to tackle three important issues in eQTL mapping. First, increasing studies have considered the genetic control of dynamic gene expression during cell and organ development^6^. Functional clustering, aimed to classify gene expression profiles based on their dynamic changes using parametric or nonparametric approaches^27^,^28^,^29^, can be integrated with the block mixture model, which allows dynamic eQTLs for gene clustering to be characterized. Second, to study how the organism responds to changing environment, gene expression experiments frequently include multiple environments or multiple tissues^24^,^25^,^26^. The implementation of the block mixture model with multiple environments enables us to understand the impact of genotype-environment interactions on regulatory machineries. Third, eQTL mapping with single or pairs of markers may confound the effects of individual eQTLs that are linked with other eQTLs. High-dimensional variable selection approaches have been developed to analyze the genetic association of a phenotypic trait with a high-dimensional set of markers at the same time through penalized regression^30^,^31^,^32^. By incorporating these approaches into the block mixture model, we will be in an excellent position to draw a precise picture of the genetic architecture of gene expression contributed by eQTLs.

## Methods

In the supplementary text, we provide the details on the derivation and implementation of a block mixture used to map eQTLs for gene clustering. Here, we describe the basic procedure of the model derivation which can be understood without heavy statistical knowledge. From this derivation we build up a joint framework that integrates gene clustering and eQTL mapping for a mapping population.

### Data structure

The data for the joint clustering and mapping framework construction is characterized by an *n*** × ***m* matrix, expressed as


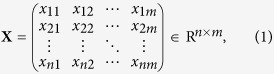


where *x*_*ij*_ denotes the gene expression level of individual *i* from a mapping population on transcript *j*. The data **X** contains two independent latent variables, i.e., the row label 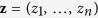 and the column label 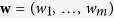. As the latent variable of gene transcripts, **w** is composed of *w*_*j*,_ the label variable of column or gene *j* (*j* = 1, …, *m*), being i.i.d. from gene cluster 1 to *L* with probability 
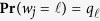
. Representing the latent genotype variable of individuals, **z** is clustered on a given eQTL *ζ*, bracketed by two flanking markers *η* and *η* + 1. The row label 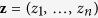 in terms of *ζ* ’s *K* latent genotypes will depend on the genotypes of the flanking markers and the position of *ζ* within the marker interval, with probability for individual *i* to carry the *k*th genotype, expressed as 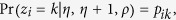 where *ρ* measures the eQTL’s position, described by the recombination fractions between the two markers and eQTL.

### Composite likelihood

We assume normal mixture components for DNA microarray. Specifically, we assume that given the row label **z** and column label **w**, data 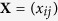 are independent with univariate normal distribution 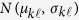. Let 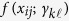 denote the corresponding density functions, where 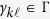. Then, the likelihood function


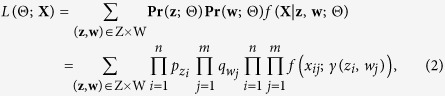


where Z and W denote the sets of all possible combinations of **z** and **w**, and 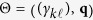 contains the unknown parameters to be estimated (in practice we fix *p*_*ik*_ so that **Θ** does not contain *p*_*ik*_). The corresponding composite likelihood function can be written as


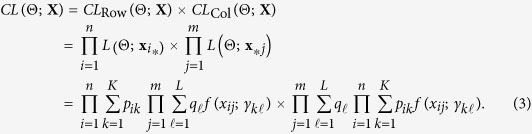


Under the setting of a block mixture model, both rows and columns are exchangeable but not independent, which characterizes the form of dependence among transcripts.

### Estimation via the EM algorithm

The maximum composite likelihood estimation of Θ can be estimated by a similar two-layer EM algorithm described in the supplementary text. In the E step, we calculate the weights in the surrogate function at iteration *t*, expressed as


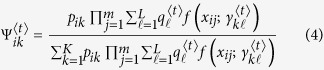



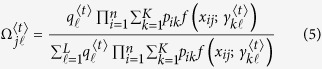



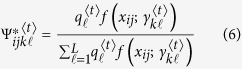



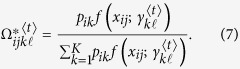


In the M step, we can further maximize the surrogate function from the estimated weights 
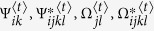
 in the E step. The basic optimization techniques lead to


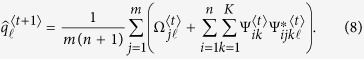


and


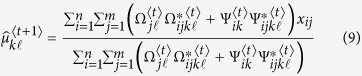



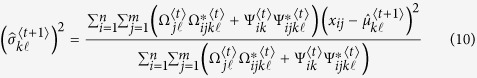


In eQTL interval mapping, the number of row components is fixed for a specific mapping population derived from a controlled cross, but the number of column components needs to be determined. By fixing the number of row components, we first implement the composite likelihood BIC to determine the optimal number of column components, from which we integrate interval mapping to map eQTLs through the entire genetic linkage map.

### Hypothesis tests

After the maximum composite likelihood of the parameters are obtained, biologically meaningful hypotheses can be tested on the basis of the composite log-likelihood ratio test statistic. Here, we consider only a simple backcross design, i.e., *K* = 2. These hypothesis tests include

1. Testing whether an eQTL affects the expression level of at least one cluster, which can be formulated as


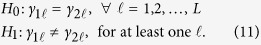


If the *H*_0_ is rejected, it means the the eQTL significantly affects the expression of all clusters.

2. Testing whether an eQTL affects the expression level of a given gene cluster. For a cluster 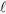, the hypothesis can be formulated as


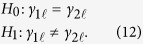


If the *H*_0_ is rejected, it means the eQTL significantly affects the expression of this particular cluster.

3. Testing whether an eQTL affects cluster-dependent difference in gene expression, which can be formulated as


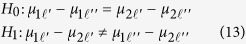


If the *H*_0_ is rejected, it means the eQTL significantly affects the difference of gene expression between different clusters.

4.Testing whether an eQTL affects cluster-structure, which can be formulated as


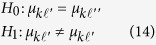


If the *H*_0_ is rejected, it means the eQTL significantly affects the gene expression between different clusters in the same genotype.

The test statistics for hypothesis tests (11)–(14) are calculated as the log composite likelihood ratio of the full over reduced model:


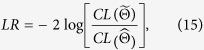


where 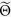 and 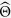 denote the maximum composite likelihood estimates under the *H*_0_ and *H*_1_, respectively. Permutation tests are used to determine the empirical genome-wide critical threshold.

## Additional Information

**How to cite this article**: Wang, N. *et al.* A block mixture model to map eQTLs for gene clustering and networking. *Sci. Rep.* **6**, 21193; doi: 10.1038/srep21193 (2016).

## Supplementary Material

Supplementary Information

## References

[b1] Mackay T. F. The genetic architecture of quantitative traits. Annu. Rev. Genet. 35, 303–339 (2001). 11700286 10.1146/annurev.genet.35.102401.090633

[b2] Schadt E. E. *et al.* An integrative genomics approach to infer causal associations between gene expression and disease. Nat. Genet. 37, 710–717 (2005). 15965475 10.1038/ng1589 PMC2841396

[b3] Emilsson V. *et al.* Genetics of gene expression and its effect on disease. Nature 452, 423–428 (2008). 18344981 10.1038/nature06758

[b4] Cookson W., Liang L., Abecasis G., Moffatt M. & Lathrop M. Mapping complex disease traits with global gene expression. Nat. Rev. Genet. 10, 184–194 (2009). 19223927 10.1038/nrg2537 PMC4550035

[b5] Rockman M. V., Skrovanek S. S. & Kruglyak L. Selection at linked sites shapes heritable phenotypic variation in C. elegans. Science 330, 372–376 (2010). 20947766 10.1126/science.1194208 PMC3138179

[b6] Francesconi M. & Lehner B. The effects of genetic variation on gene expression dynamics during development. Nature 505, 208–211 (2014). 24270809 10.1038/nature12772

[b7] Kim Y. *et al.* A meta-analysis of gene expression quantitative trait loci in brain. Transl. psychiatry 4, e459 (2014). 25290266 10.1038/tp.2014.96 PMC4350525

[b8] Fairfax B. P. *et al.* Innate immune activity conditions the effect of regulatory variants upon monocyte gene expression. Science 343, 1246949 (2014). 24604202 10.1126/science.1246949 PMC4064786

[b9] Lee M. N. *et al.* Common genetic variants modulate pathogen-sensing responses in human dendritic cells. Science 343, 1246980 (2014). 24604203 10.1126/science.1246980 PMC4124741

[b10] Govaert G. & Nadif M. Clustering with block mixture models. Pattern Recognit. 36, 463–473 (2003).

[b11] Govaert G. & Nadif M. An EM algorithm for the block mixture model. IEEE Trans. Pattern Anal. Mach. Intell. 27, 643–647 (2005). 15794169 10.1109/TPAMI.2005.69

[b12] Govaert G. & Nadif M. Block clustering with bernoulli mixture models: Comparison of different approaches. Comput. Stat. Data Anal. 52, 3233–3245 (2008).

[b13] Kuruppumullage Don P. Estimation and model selection for block clustering with mixtures: A composite likelihood approach (PhD diss., PSU, 2014).

[b14] Chun H. & Keleş S. Expression quantitative trait loci mapping with multivariate sparse partial least squares regression. Genetics 182, 79–90 (2009). 19270271 10.1534/genetics.109.100362 PMC2674843

[b15] Kendziorski C. M., Chen M., Yuan M., Lan H. & Attie A. D. Statistical methods for expression quantitative trait loci (eQTL) mapping. Biometrics 62, 19–27 (2006). 16542225 10.1111/j.1541-0420.2005.00437.x

[b16] Arbeitman M. N. *et al.* Gene expression during the life cycle of Drosophila melanogaster. Science 297, 2270–2275 (2002). 12351791 10.1126/science.1072152

[b17] D’haeseleer P. How does gene expression clustering work ? Nat. Biotechnol. 23, 1499–1501 (2005). 16333293 10.1038/nbt1205-1499

[b18] De Smet R. & Marchal K. Advantages and limitations of current network inference methods. Nat. Rev. Microbiol. 8, 717–729 (2010). 20805835 10.1038/nrmicro2419

[b19] Marbach D. *et al.* Wisdom of crowds for robust gene network inference. Nat. Methods 9, 796–804 (2012). 22796662 10.1038/nmeth.2016 PMC3512113

[b20] Brem R. B., Yvert G., Clinton R. & Kruglyak L. Genetic dissection of transcriptional regulation in budding yeast. Science 296, 752–755 (2002). 11923494 10.1126/science.1069516

[b21] Rockman M. V. & Kruglyak L. Genetics of global gene expression. Nat. Rev. Genet. 7, 862–872 (2006). 17047685 10.1038/nrg1964

[b22] Viñuela A., Snoek L. B., Riksen J. A. G. & Kammenga J. E. Genome-wide gene expression regulation as a function of genotype and age in C. elegans. Genome Res. 20, 929–937 (2010). 20488933 10.1101/gr.102160.109 PMC2892094

[b23] Ackermann M., Sikora-Wohlfeld W. & Beyer A. Impact of natural genetic variation on gene expression dynamics. PLoS Genet. 9, e1003514 (2013). 23754949 10.1371/journal.pgen.1003514 PMC3674999

[b24] Wang N. *et al.* A bi-Poisson model for clustering gene expression profiles by RNA-seq. Brief. Bioinform. 15, 534–541 (2014). 23665510 10.1093/bib/bbt029 PMC4192042

[b25] Jiang L., Mao K. & Wu R. A skellam model to identify differential patterns of gene expression induced by environmental signals. BMC Genomics 15, 772 (2014). 25199446 10.1186/1471-2164-15-772 PMC4167515

[b26] Ye M., Wang Z., Wang Y. & Wu R. A multi-Poisson dynamic mixture model to cluster developmental patterns of gene expression by RNA-seq. Brief. Bioinform. 16, 205–215 (2015). 24817567 10.1093/bib/bbu013

[b27] Kim B. R., Zhang L., Berg A., Fan J. & Wu R. A computational approach to the functional clustering of periodic gene-expression profiles. Genetics 180, 821–834 (2008). 18780724 10.1534/genetics.108.093690 PMC2567383

[b28] Kim B. R., McMurry T., Zhao W., Wu R. & Berg A. Wavelet-based functional clustering for patterns of high-dimensional dynamic gene expression. J. Comp. Biol. 17, 1067–1080 (2010). 10.1089/cmb.2009.0270 PMC3133835 20726793

[b29] Li N. *et al.* Functional clustering of periodic transcriptional profiles through ARMA (p, q). PloS One 5, e9894 (2010). 20419127 10.1371/journal.pone.0009894 PMC2855703

[b30] Li J., Das K., Fu G., Li R. & Wu R. The Bayesian lasso for genome-wide association studies. Bioinformatics 27, 516–523 (2011). 21156729 10.1093/bioinformatics/btq688 PMC3105480

[b31] Li J., Zhong W., Li R. & Wu R. A fast algorithm for detecting gene–gene interactions in genome-wide association studies. Ann. Appl. Stat. 8, 2292–2318 (2014). 26457126 10.1214/14-aoas771 PMC4595934

[b32] Li J., Zhong W., Li R. & Wu R. Bayesian group LASSO for nonparametric varying-coefficient models with application to functional genome-wide association studies. Ann. Appl. Stat. 9, 640–664 (2015). 26478762 10.1214/15-AOAS808 PMC4605444

