# Supplementary material for: A block mixture model to map eQTLs for gene clustering and networking
Source: Sci Rep. 2016 Feb 19;6:21193. doi: 10.1038/srep21193 (PMC4759821; doi:10.1038/srep21193)
Supplement: Supplementary Information [file srep21193-s1.pdf]

Supplementary Information

A block mixture model to map eQTLs for gene clustering and networking

Ningtao Wang[1], Kirk Gosik[2], Runze Li[2,3], Bruce Lindsay[3], Rongling Wu[2,3,*]

* Corresponding auther

Email: rwu@hes.hmc.psu.edu

[1]Department of Biostatistics, University of Texas School of Public Health, Houston, TX 77030, USA

[2]Department of Public Health Sciences, The Pennsylvania State University, Hershey, PA 17033, USA

[3]Department of Statistics, The Pennsylvania State University, University Park, PA 16802, USA

Supplementary Table 1: Results of parameter estimates from simulated data by mimicking the data structure of C. elegan data. The maximum composite likelihood estimates (MCLEs) from the model are compared with the true value. The standard errors of the MCLEs (in parentheses) are calculated from 500 replicates.

Cluster	Proportion		μ_1		μ_2		σ_1		σ_2	
	True	MCLE	True	MCLE	True	MCLE	True	MCLE	True	MCLE
1	0.0141	0.0140	-2.771	-2.763	0.031	0.026	1.458	1.463	0.423	0.444
		(6.0e-4)		(0.029)		(0.012)		(0.016)		(0.044)
2	0.0597	0.0605	-2.176	-2.169	0.440	0.435	0.581	0.592	0.250	0.271
		(0.0019)		(0.016)		(0.011)		(0.022)		(0.045)
3	6.1e-4	6.0e-4	-4.220	-4.198	2.731	2.659	1.683	1.719	2.581	2.565
		(7.1e-5)		(0.114)		(0.218)		(0.093)		(0.151)
4	0.0021	0.0020	-3.330	-3.313	2.186	2.172	0.761	0.808	1.779	1.787
		(1.6e-4)		(0.039)		(0.072)		(0.092)		(0.051)
5	0.0147	0.0145	-0.998	-0.997	-0.004	-0.005	1.455	1.455	0.470	0.476
		(3.7e-4)		(0.019)		(0.006)		(0.012)		(0.014)
6	0.0027	0.0027	-2.855	-2.843	2.027	2.010	1.448	1.468	1.899	1.907
		(1.7e-4)		(0.047)		(0.068)		(0.048)		(0.042)
7	0.0068	0.0067	-2.570	-2.555	2.078	2.067	0.806	0.836	1.275	1.288
		(3.1e-4)		(0.032)		(0.038)		(0.059)		(0.032)
8	0.0369	0.0371	-1.596	-1.591	1.124	1.119	0.663	0.675	0.908	0.915
		(7.4e-4)		(0.012)		(0.013)		(0.022)		(0.016)
9	0.0419	0.0423	-1.355	-1.351	0.893	0.889	0.444	0.456	0.763	0.769
		(0.0011)		(0.009)		(0.010)		(0.024)		(0.014)
10	0.0341	0.0345	-1.215	-1.212	0.777	0.774	0.321	0.333	0.674	0.679
		(0.0011)		(0.006)		(0.009)		(0.023)		(0.012)
11	0.0545	0.0544	-1.057	-1.056	0.659	0.656	0.376	0.385	0.602	0.606
		(0.0011)		(0.003)		(0.008)		(0.017)		(0.010)
12	0.0139	0.0138	-0.910	-0.909	0.679	0.676	0.282	0.293	0.714	0.717
		(4.2e-4)		(0.004)		(0.010)		(0.021)		(0.009)
13	0.0145	0.0144	-1.946	-1.937	1.766	1.757	0.893	0.909	1.036	1.046
		(4.0e-4)		(0.021)		(0.025)		(0.030)		(0.022)
14	0.0279	0.0283	-1.525	-1.519	1.347	1.342	0.473	0.491	0.663	0.674
		(8.3e-4)		(0.011)		(0.012)		(0.035)		(0.025)
15	0.0324	0.0319	0.002	0.002	-0.165	-0.165	1.136	1.138	0.863	0.865
		(0.0012)		(0.009)		(0.007)		(0.008)		(0.006)
16	0.0055	0.0055	-0.069	-0.071	-0.075	-0.076	3.238	3.224	0.715	0.726
		(1.8e-4)		(0.061)		(0.012)		(0.055)		(0.029)
17	0.0174	0.0174	-2.249	-2.236	2.115	2.105	0.671	0.700	0.872	0.889
		(6.2e-4)		(0.026)		(0.027)		(0.055)		(0.037)
18	0.0417	0.0422	-1.768	-1.760	1.670	1.663	0.564	0.585	0.708	0.722
		(0.0013)		(0.016)		(0.016)		(0.040)		(0.030)
19	0.0170	0.0171	1.165	1.161	-1.257	-1.253	0.465	0.478	0.427	0.439
		(4.5e-4)		(0.009)		(0.009)		(0.025)		(0.027)
20	0.0182	0.0181	1.000	0.998	-1.056	-1.053	0.832	0.837	0.818	0.823
		(3.9e-4)		(0.010)		(0.012)		(0.012)		(0.012)
21	0.0298	0.0295	-0.926	-0.924	0.871	0.867	0.369	0.378	0.405	0.413
		(0.0010)		(0.004)		(0.009)		(0.018)		(0.017)
22	0.0265	0.0263	0.879	0.879	-0.923	-0.920	0.653	0.658	0.668	0.673
		(6.3e-4)		(0.007)		(0.009)		(0.011)		(0.012)
23	0.0090	0.0089	0.023	0.022	-0.042	-0.042	2.506	2.503	0.394	0.403
		(2.8e-4)		(0.039)		(0.006)		(0.027)		(0.022)
24	0.0326	0.0328	1.947	1.936	-1.954	-1.944	0.747	0.766	0.635	0.654
		(9.0e-4)		(0.022)		(0.022)		(0.038)		(0.041)
25	0.0064	0.0063	1.952	1.941	-1.920	-1.905	1.323	1.334	1.343	1.353
		(2.5e-4)		(0.031)		(0.038)		(0.027)		(0.028)
26	0.0221	0.0217	0.062	0.062	-0.022	-0.021	1.496	1.496	0.356	0.360
		(6.4e-4)		(0.012)		(0.004)		(0.011)		(0.011)
27	0.0140	0.0138	0.097	0.118	-0.032	-0.040	1.652	1.654	0.603	0.605
		(3.5e-4)		(0.050)		(0.019)		(0.016)		(0.007)
28	0.0224	0.0224	1.052	1.049	-0.978	-0.975	0.479	0.488	0.513	0.521
		(4.6e-4)		(0.007)		(0.008)		(0.018)		(0.017)

29	0.0266	0.0268	1.343	1.338	-1.174	-1.171	0.532	0.545	0.639	0.648
		(6.8e-4)		(0.010)		(0.009)		(0.024)		(0.020)
30	0.0115	0.0114	0.458	0.459	-0.276	-0.274	1.478	1.479	1.363	1.363
		(3.4e-4)		(0.019)		(0.019)		(0.013)		(0.011)
31	0.0263	0.0260	0.173	0.173	0.010	0.011	1.261	1.263	1.083	1.085
		(8.7e-4)		(0.010)		(0.009)		(0.008)		(0.007)
32	0.0408	0.0401	0.175	0.176	0.045	0.045	1.099	1.102	0.843	0.844
		(0.0015)		(0.008)		(0.006)		(0.007)		(0.006)
33	0.0243	0.0241	0.903	0.902	-0.675	-0.673	0.534	0.539	0.601	0.605
		(5.0e-4)		(0.006)		(0.008)		(0.010)		(0.011)
34	0.0187	0.0186	1.548	1.541	-1.303	-1.296	1.089	1.095	1.159	1.164
		(3.8e-4)		(0.018)		(0.022)		(0.015)		(0.013)
35	0.0351	0.0355	1.609	1.603	-1.355	-1.349	0.575	0.591	0.771	0.780
		(0.0010)		(0.014)		(0.013)		(0.031)		(0.020)
36	0.0161	0.0162	1.225	1.222	-0.850	-0.846	0.577	0.584	0.740	0.746
		(3.4e-4)		(0.009)		(0.011)		(0.014)		(0.013)
37	0.0239	0.0240	1.458	1.453	-1.036	-1.033	0.746	0.753	0.889	0.894
		(4.1e-4)		(0.012)		(0.014)		(0.016)		(0.014)
38	0.0038	0.0038	0.846	1.131	-0.328	-0.368	1.645	1.686	0.446	0.452
		(9.6e-5)		(0.605)		(0.083)		(0.095)		(0.023)
39	0.0300	0.0302	1.890	1.880	-1.364	-1.357	0.702	0.717	0.936	0.944
		(6.6e-4)		(0.019)		(0.018)		(0.030)		(0.019)
40	0.0413	0.0419	1.189	1.185	-0.564	-0.562	0.269	0.282	0.777	0.780
		(0.0018)		(0.007)		(0.009)		(0.025)		(0.010)
41	0.0221	0.0218	1.785	1.776	-0.275	-0.271	1.253	1.255	0.355	0.369
		(6.1e-4)		(0.021)		(0.010)		(0.011)		(0.033)
42	0.0162	0.0158	2.431	2.418	-0.591	-0.588	1.877	1.878	0.170	0.180
		(8.2e-4)		(0.036)		(0.005)		(0.022)		(0.023)
43	0.0438	0.0441	2.297	2.290	0.006	0.011	0.428	0.440	0.424	0.435
		(0.0017)		(0.014)		(0.012)		(0.024)		(0.024)

Supplementary Table 2: Gene enrichment analysis by using PANTHER overrepresentation test. Whose p-values are smaller than 1e-10 after Bonferroni correction are listed below.

Cluster	Size	GO slim biological process
8	190	primary metabolic process
		metabolic process
		protein metabolic process
9	236	primary metabolic process
		metabolic process
		protein metabolic process
		nucleobase-containing compound metabolic process
10	185	metabolic process
		primary metabolic process
		nucleobase-containing compound metabolic process
		protein metabolic process
		RNA metabolic process
11	331	primary metabolic process
		metabolic process
		nucleobase-containing compound metabolic process
		protein metabolic process
		RNA metabolic process
		biological regulation
		transcription, DNA-dependent
		transcription from RNA polymerase II promoter
		translation
12	143	metabolic process
		primary metabolic process
		cellular process
		protein metabolic process
13	186	metabolic process
14	182	metabolic process
		primary metabolic process
		cellular process
17	250	metabolic process
18	317	metabolic process
		primary metabolic process
		cellular process
		nucleobase-containing compound metabolic process
20	167	metabolic process
21	227	metabolic process
		primary metabolic process
		nucleobase-containing compound metabolic process
22	253	primary metabolic process
		protein metabolic process
		metabolic process
		cellular process
		localization
		transport
		cellular component organization or biogenesis
24	253	metabolic process
		primary metabolic process
		protein metabolic process
		cellular process
28	210	metabolic process
		primary metabolic process
33	219	metabolic process
		primary metabolic process
		protein metabolic process
35	137	metabolic process
		primary metabolic process
36	145	metabolic process
		primary metabolic process
		cellular process
37	171	metabolic process
		primary metabolic process
39	143	primary metabolic process

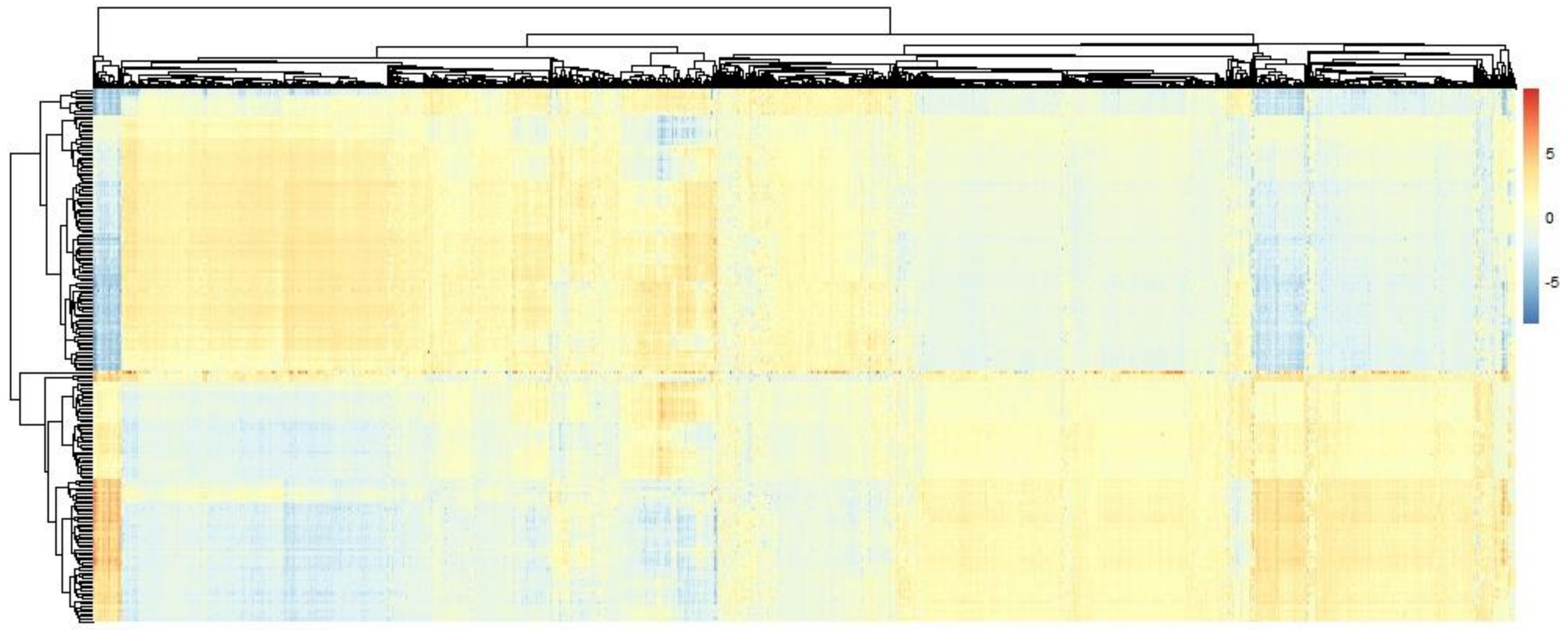

Supplementary Figure 1: Heatmap of *C. elegans* gene expression data

Supplementary Text: Block Mixture

Model Derivations

Block Mixture of Univariate Normal Components.

For a continuous array data $\mathbf{X} \in \mathrm{R}^{n \times m}$, we assume there are two independent latent variables, the row label variable $\mathbf{z} = (z_1, \ldots, z_n)$ and the column label variable $\mathbf{w} = (w_1, \ldots, w_m)$. Here, z_i is the label variable for row *i*, and $z_1, \ldots, z_n$ are independent and identically distributed (i.i.d.) from 1 to *K* with probability $\Pr(z_i = k) = p_k$. Similarly, w_j is the label variable for column *j*, and $w_1, \ldots, w_m$ are i.i.d. from 1 to *L* with probability $\Pr(w_j = \ell) = q_\ell$. By the setting, the row and column label variables z and w are independent, which matches the row column independent clustering problems in computer science and is named as "block mixture model" in Govaert and Nadif (2003, 2005, 2008).

Composite Likelihood.

We assume that given the row label **z** and column label w, the data $\mathbf{X} = (x_{ij})$ are independent with normal distribution $N(\mu_{k\ell}, \sigma_{k\ell}^2)$. Let $f(x_{ij}; \gamma_{k\ell})$ denote the corresponding density functions, where $\gamma_{k\ell} \in \Gamma$. Then, the full likelihood function

$$\begin{aligned} L(\Theta; \mathbf{X}) &= \sum_{(\mathbf{z},\mathbf{w}) \in \mathbb{Z} \times \mathbb{W}} \Pr(\mathbf{z}; \Theta) \Pr(\mathbf{w}; \Theta) f(\mathbf{X} \mid \mathbf{z}, \mathbf{w}; \Theta) \\ &= \sum_{(\mathbf{z},\mathbf{w}) \in \mathbb{Z} \times \mathbb{W}} \prod_{i=1}^{n} p_{z_i} \prod_{j=1}^{m} q_{w_j} \prod_{i=1}^{n} \prod_{j=1}^{m} f(x_{ij}; \gamma(z_i, w_j)), \end{aligned} \tag{1}$$

where **Z** and **W** denote the sets of all possible combinations of **z** and **w**, and $\Theta = ((\gamma_{k\ell}), \mathbf{p}, \mathbf{q})$ contains the unknown parameters to be estimated.

A one-time naive calculation of the full likelihood function has huge computational burden. The number of sums in (1) is at least exponential in $\min(m, n)$, and far exceeds the modern

computation capability. One solution is proposed by using the composite likelihood as an approximation of the original likelihood function (1) (Kuruppumullage Don 2014). We here consider the composite likelihood

$$\begin{aligned} CL(\Theta;\mathbf{X}) &= CL_{\text{Row}}(\Theta;\mathbf{X}) \times CL_{\text{Col}}(\Theta;\mathbf{X}) \\ &= \prod_{i=1}^{n} L(\Theta;\mathbf{x}_{i*}) \times \prod_{j=1}^{m} L(\Theta;\mathbf{x}_{*j}) \\ &= \prod_{i=1}^{n} \sum_{k=1}^{K} p_k \prod_{j=1}^{m} \sum_{\ell=1}^{L} q_\ell f(x_{ij};\gamma_{k\ell}) \times \prod_{j=1}^{m} \sum_{\ell=1}^{L} q_\ell \prod_{i=1}^{n} \sum_{k=1}^{K} p_k f(x_{ij};\gamma_{k\ell}), \end{aligned} \tag{2}$$

Here $\mathbf{x}_{i*}$ and $\mathbf{x}_{*j}$ are *i*th row and *j*th column of **X**. Composite likelihood is the likelihood type of object formed by multiplying individual component likelihood, each of which corresponds to a margin or conditional event (Lindsay 1988). The proposed composite likelihoods (2) are constructed by single row and column likelihood, which assumes both of them are pseudo-independent. Composite likelihood guarantees the unbiased estimation equations for the original parameters. Comparing with the original likelihood method, the composite likelihood method is less statistically efficient, but can dramatically decrease the computational price (Lindsay et al. 2011).

Estimation via EM Algorithms.

The maximum composite likelihood estimates of Θ is proposed to be estimated via a two-layer EM algorithm in Kuruppumullage Don (2014). In the E step, to build the algorithm, we create the surrogate function of Θ given $\Theta^{\langle t \rangle}$, by using standard EM derivation twice. Firstly, the log composite likelihood is

$$\begin{aligned} \log CL(\Theta;\mathbf{X}) &= \log CL_{\text{Row}}(\Theta;\mathbf{X}) + \log CL_{\text{Col}}(\Theta;\mathbf{X}) \\ &= \sum_{i=1}^{n} \log\left\{ \sum_{k=1}^{K} p_k \prod_{j=1}^{m} \sum_{\ell=1}^{L} q_\ell f(x_{ij};\gamma_{k\ell}) \right\} \\ &+ \sum_{j=1}^{m} \log\left\{ \sum_{\ell=1}^{L} q_\ell \prod_{i=1}^{n} \sum_{k=1}^{K} p_k f(x_{ij};\gamma_{k\ell}) \right\}. \end{aligned}$$

Secondly, we implement the standard EM trick to get the logarithm inside the sum, and obtain the first surrogate function,

$$\begin{aligned}
Q^*(\Theta \mid \Theta^{\langle t \rangle}) &= \sum_{i=1}^{n}\sum_{k=1}^{K}\Psi_{ik}^{\langle t \rangle} \log\left[p_k \prod_{j=1}^{m}\sum_{\ell=1}^{L} q_\ell f(x_{ij};\gamma_{k\ell}) \right] \\
&\quad + \sum_{j=1}^{m}\sum_{\ell=1}^{L}\Omega_{j\ell}^{\langle t \rangle} \log\left[q_\ell \prod_{i=1}^{n}\sum_{k=1}^{K} p_k f(x_{ij};\gamma_{k\ell}) \right] \\
&= \sum_{i=1}^{n}\sum_{k=1}^{K}\Psi_{ik}^{\langle t \rangle} \log p_k + \sum_{i=1}^{n}\sum_{k=1}^{K}\Psi_{ik}^{\langle t \rangle} \sum_{j=1}^{m} \log\left[\sum_{\ell=1}^{L} q_\ell f(x_{ij};\gamma_{k\ell}) \right] \\
&\quad + \sum_{j=1}^{m}\sum_{\ell=1}^{L}\Omega_{j\ell}^{\langle t \rangle} \log q_\ell + \sum_{j=1}^{m}\sum_{\ell=1}^{L}\Omega_{j\ell}^{\langle t \rangle} \sum_{i=1}^{n} \log\left[\sum_{k=1}^{K} p_k f(x_{ij};\gamma_{k\ell}) \right],
\end{aligned}$$

where

$$\Psi_{ik}^{\langle t \rangle} = \frac{p_k^{\langle t \rangle} \prod_{j=1}^{m}\sum_{\ell=1}^{L} q_\ell^{\langle t \rangle} f(x_{ij};\gamma_{k\ell}^{\langle t \rangle})}{\sum_{k=1}^{K} p_k^{\langle t \rangle} \prod_{j=1}^{m}\sum_{\ell=1}^{L} q_\ell^{\langle t \rangle} f(x_{ij};\gamma_{k\ell}^{\langle t \rangle})}$$

$$\Omega_{j\ell}^{\langle t \rangle} = \frac{q_\ell^{\langle t \rangle} \prod_{i=1}^{n}\sum_{k=1}^{K} p_k^{\langle t \rangle} f(x_{ij};\gamma_{k\ell}^{\langle t \rangle})}{\sum_{\ell=1}^{L} q_\ell^{\langle t \rangle} \prod_{i=1}^{n}\sum_{k=1}^{K} p_k^{\langle t \rangle} f(x_{ij};\gamma_{k\ell}^{\langle t \rangle})}.$$

Finally, we apply the EM trick again to move the last sum outside the logarithm, and obtain the final surrogate function,

$$\begin{aligned}
Q(\Theta \mid \Theta^{\langle t \rangle}) &= \sum_{i=1}^{n}\sum_{k=1}^{K}\Psi_{ik}^{\langle t \rangle} \log p_k + \sum_{i=1}^{n}\sum_{k=1}^{K}\Psi_{ik}^{\langle t \rangle} \sum_{j=1}^{m}\sum_{\ell=1}^{L}\Psi_{ijk\ell}^{*\langle t \rangle} \log\left(q_\ell f(x_{ij};\gamma_{k\ell}) \right) \\
&\quad + \sum_{j=1}^{m}\sum_{\ell=1}^{L}\Omega_{j\ell}^{\langle t \rangle} \log q_\ell + \sum_{j=1}^{m}\sum_{\ell=1}^{L}\Omega_{j\ell}^{\langle t \rangle} \sum_{i=1}^{n}\sum_{k=1}^{K}\Omega_{ijk\ell}^{*\langle t \rangle} \log\left(p_k f(x_{ij};\gamma_{k\ell}) \right) \\
&= \sum_{i=1}^{n}\sum_{k=1}^{K}\left(\Psi_{ik}^{\langle t \rangle} + \sum_{j=1}^{m}\sum_{\ell=1}^{L}\Omega_{j\ell}^{\langle t \rangle}\Omega_{ijk\ell}^{*\langle t \rangle} \right) \log p_k \\
&\quad + \sum_{j=1}^{m}\sum_{\ell=1}^{L}\left(\Omega_{j\ell}^{\langle t \rangle} + \sum_{i=1}^{n}\sum_{k=1}^{K}\Psi_{ik}^{\langle t \rangle}\Psi_{ijk\ell}^{*\langle t \rangle} \right) \log q_\ell \\
&\quad + \sum_{i=1}^{n}\sum_{j=1}^{m}\sum_{k=1}^{K}\sum_{\ell=1}^{L}\left(\Omega_{j\ell}^{\langle t \rangle}\Omega_{ijk\ell}^{*\langle t \rangle} + \Psi_{ik}^{\langle t \rangle}\Psi_{ijk\ell}^{*\langle t \rangle} \right) \log f(x_{ij};\gamma_{k\ell}),
\end{aligned}$$

where

$$\Psi_{ijk\ell}^{*\langle t\rangle} = \frac{q_\ell^{\langle t\rangle} f(x_{ij};\gamma_{k\ell}^{\langle t\rangle})}{\sum_{\ell=1}^{L} q_\ell^{\langle t\rangle} f(x_{ij};\gamma_{k\ell}^{\langle t\rangle})}$$

$$\Omega_{ijk\ell}^{*\langle t\rangle} = \frac{p_k^{\langle t\rangle} f(x_{ij};\gamma_{k\ell}^{\langle t\rangle})}{\sum_{k=1}^{K} p_k^{\langle t\rangle} f(x_{ij};\gamma_{k\ell}^{\langle t\rangle})}.$$

In the M step, once the weights $\Psi_{ik}^{\langle t\rangle}, \Psi_{ijk\ell}^{*\langle t\rangle}, \Omega_{j\ell}^{\langle t\rangle}, \Omega_{ijk\ell}^{*\langle t\rangle}$ are obtained, we can further maximize the surrogate function, and map $\Theta^{\langle t\rangle}$ to $\Theta^{\langle t+1\rangle}$. The basic optimization techniques lead to

$$\hat{p}_k^{\langle t+1\rangle} = \frac{1}{n(m+1)} \sum_{i=1}^{n} \left(\Psi_{ik}^{\langle t\rangle} + \sum_{j=1}^{m}\sum_{\ell=1}^{L} \Omega_{j\ell}^{\langle t\rangle} \Omega_{ijk\ell}^{*\langle t\rangle} \right)$$

$$\hat{q}_\ell^{\langle t+1\rangle} = \frac{1}{m(n+1)} \sum_{j=1}^{m} \left(\Omega_{j\ell}^{\langle t\rangle} + \sum_{i=1}^{n}\sum_{k=1}^{K} \Psi_{ik}^{\langle t\rangle} \Psi_{ijk\ell}^{*\langle t\rangle} \right).$$

and

$$\hat{\mu}_{k\ell}^{\langle t+1\rangle} = \frac{\sum_{i=1}^{n}\sum_{j=1}^{m} \left(\Omega_{j\ell}^{\langle t\rangle}\Omega_{ijk\ell}^{*\langle t\rangle} + \Psi_{ik}^{\langle t\rangle}\Psi_{ijk\ell}^{*\langle t\rangle} \right) x_{ij}}{\sum_{i=1}^{n}\sum_{j=1}^{m} \left(\Omega_{j\ell}^{\langle t\rangle}\Omega_{ijk\ell}^{*\langle t\rangle} + \Psi_{ik}^{\langle t\rangle}\Psi_{ijk\ell}^{*\langle t\rangle} \right)}$$

$$\left(\hat{\sigma}_{k\ell}^{\langle t+1\rangle}\right)^2 = \frac{\sum_{i=1}^{n}\sum_{j=1}^{m} \left(\Omega_{j\ell}^{\langle t\rangle}\Omega_{ijk\ell}^{*\langle t\rangle} + \Psi_{ik}^{\langle t\rangle}\Psi_{ijk\ell}^{*\langle t\rangle} \right) \left(x_{ij} - \hat{\mu}_{k\ell}^{\langle t+1\rangle} \right)^2}{\sum_{i=1}^{n}\sum_{j=1}^{m} \left(\Omega_{j\ell}^{\langle t\rangle}\Omega_{ijk\ell}^{*\langle t\rangle} + \Psi_{ik}^{\langle t\rangle}\Psi_{ijk\ell}^{*\langle t\rangle} \right)}.$$

Clustering.

Once the block mixture model has been fitted, the question of latent class estimation arises, and it is related to row column independent clustering problem. Based on the Bayes rule, the assignment of row i to row components is achieved by assigning it to the the component to which it has the highest estimated posterior probability of belonging,

$$\begin{aligned} \tilde{z}_i &= \arg\max_k \Pr(z_i = k \mid \mathbf{X}, \hat{\Theta}) \\ &= \arg\max_k \left\{ \frac{\Pr(z_i = k, \mathbf{X} \mid \hat{\Theta})}{\Pr(\mathbf{X} \mid \hat{\Theta})} \right\}. \end{aligned}$$

However, it is equally complex to be calculated as the original likelihood function $L(\Theta;\mathbf{X})$ (Kuruppumullage Don 2014).

When we construct the composite likelihood, it is assumed that both rows and columns are pseudo-independent. Under such an independent assumption $\Pr(z_i = k \mid \mathbf{X}) = \Pr(z_i = k \mid \mathbf{x}_{i*})$ where $\mathbf{x}_{i*}$ is the i th row in $\mathbf{X}$. Moveover, the proposed two-layer EM algorithm can obtain the weights $\Psi_{ik}^{\langle t \rangle} = \Pr(z_i = k \mid \mathbf{x}_{i*}, \hat{\Theta}^{\langle t \rangle})$ in each E step. Hence, it is natural and convenient to assign z_i by its posterior probability conditioning on row i instead of conditioning on the entire array data, and then

$$\hat{z}_i = \arg\max_k \Pr(z_i = k \mid \mathbf{x}_{i*}, \hat{\Theta}) = \arg\max_k \Psi_{ik}.$$

A similar assignment of column j is achieved by

$$\hat{w}_j = \arg\max_\ell \Pr(w_j = k \mid \mathbf{x}_{*j}, \hat{\Theta}) = \arg\max_\ell \Omega_{j\ell},$$

where $\mathbf{x}_{*j}$ is the j th column in $\mathbf{X}$.

Model Selection.

Usually, in real data analysis, both numbers of row components and column components, i.e. K and L, need to be predetermined. In order to access the number of components, two main ways have been usually considered (McLachlan and Peel 2004). One way is based on the penalized form of the likelihood, such as Bayesian information criterion (BIC). As the likelihood increases with the number of mixture components, the log likelihood will be penalized for the number of parameters in it. The other way is to perform a hypothesis test, such as bootstrap-based likelihood ratio test (McLachlan and Peel 2004) or likelihood-based cross validation (Kuruppumullage Don 2014). However, both the bootstrap-based likelihood ratio test and the likelihood-based cross validation involve heavy computational burdens. Hence, we propose to

incorporate composite likelihood with BIC to choose both numbers of row components and column components, which is less demanding than the hypothesis test approaches.

The composite likelihood version of BIC, as given in Gao and Song (2010) by Laplace approximation, selects the model that minimizes

$$\mathrm{CL-BIC} = -2\log CL(\hat{\Theta};\mathbf{X}) + \log(N)\mathrm{tr}(\mathbf{J}(\hat{\Theta})\mathbf{H}^{-1}(\hat{\Theta})),$$

where N is number of observations,

$$\mathbf{J}(\Theta) = \mathrm{Var}_{\Theta}\left[\nabla_{\Theta}\log CL(\Theta;\mathbf{X})\right]$$

is the variability matrix and

$$\mathbf{H}(\Theta) = \mathrm{E}_{\Theta}\left[-\nabla^2_{\Theta}\log CL(\Theta;\mathbf{X})\right]$$

is the sensitivity matrix (Hessian matrix). The second Bartlett identity doesn't hold for the entire composite likelihood. Thus usually $\mathbf{J}(\hat{\Theta})$ is not equal to $\mathbf{H}(\hat{\Theta})$, and both of them have to be estimated.

For sensitivity matrix $\mathbf{H}(\hat{\Theta})$, under some regularity conditions, a consistent estimator is the negative Hessian matrix evaluated at the maximum composite likelihood estimates

$$\hat{\mathbf{H}}(\hat{\Theta}) = -\nabla^2_{\Theta}\log CL(\Theta;\mathbf{X})\Big|_{\hat{\Theta}}.$$

When Hessian matrix is difficult to calculate, an alternative estimator is

$$\hat{\mathbf{H}}(\hat{\Theta}) = \left[\sum_{i=1}^{n}\nabla_{\Theta}\log L(\mathbf{x}_{i*})\nabla_{\Theta}\log L(\mathbf{x}_{i*})^T + \sum_{j=1}^{m}\nabla_{\Theta}\log L(\mathbf{x}_{*j})\nabla_{\Theta}\log L(\mathbf{x}_{*j})^T\right]\Bigg|_{\hat{\Theta}},$$

since the second Bartlett identity remains true for both row likelihood $L(\mathbf{x}_{i*})$ and column likelihood $L(\mathbf{x}_{*j})$.

However, the efficient estimator of the variability matrix $\mathbf{J}(\hat{\Theta})$ is not straightforward, since the

associated naive estimator

$$\hat{\mathbf{J}}(\hat{\Theta}) = \{\nabla_{\Theta} \log CL(\Theta; \mathbf{X})\}\{\nabla_{\Theta} \log CL(\Theta; \mathbf{X})\}^{T}\Big|_{\hat{\Theta}}$$

vanishes when evaluated at the maximum composite likelihood estimates (Gao and Song 2010).

Here, we propose to perform simulations for the evaluation of both matrices. The matrix $\mathbf{H}(\hat{\Theta})$ has the expression

$$\begin{aligned}\mathbf{H}(\hat{\Theta}) &= \mathrm{E}_{\Theta}\Bigg[\sum_{i=1}^{n} \nabla_{\Theta} \log L(\mathbf{x}_{i*}) \nabla_{\Theta} \log L(\mathbf{x}_{i*})^{T} \\ &\quad + \sum_{j=1}^{m} \nabla_{\Theta} \log L(\mathbf{x}_{*j}) \nabla_{\Theta} \log L(\mathbf{x}_{*j})^{T}\Bigg]\Bigg|_{\hat{\Theta}},\end{aligned}$$

And the matrix $\mathbf{J}(\hat{\Theta})$ can be expressed as

$$\begin{aligned}\mathbf{J}(\hat{\Theta}) &= \mathrm{E}_{\Theta}\big[\big(\nabla_{\Theta} \log CL_{\mathrm{Row}} + \nabla_{\Theta} \log CL_{\mathrm{Col}}\big) \\ &\quad \times \big(\nabla_{\Theta} \log CL_{\mathrm{Row}} + \nabla_{\Theta} \log CL_{\mathrm{Col}}\big)^{T}\big]_{\hat{\Theta}}.\end{aligned}$$

where $\nabla_{\Theta} \log CL_{\mathrm{Col}} = \sum_{j=1}^{m} \nabla_{\Theta} \log L(\mathbf{x}_{*j})$.

Furthermore, both $\mathbf{H}(\hat{\Theta})$ and $\mathbf{J}(\hat{\Theta})$ can be estimated via simulation of the first derivative of $\log L(\mathbf{x}_{i*})$ and $\log L(\mathbf{x}_{*j})$. Specifically, the partial derivatives in $\nabla_{\Theta} \log L(\mathbf{x}_{i*})$ are

$$\frac{\partial \log L(\mathbf{x}_{i*})}{\partial p_{k^*}} = \frac{\prod_{j=1}^{m}\sum_{\ell=1}^{L} q_{\ell} f(x_{ij};\gamma_{k^*\ell}) - \prod_{j=1}^{m}\sum_{\ell=1}^{L} q_{\ell} f(x_{ij};\gamma_{K\ell})}{\sum_{k=1}^{K} p_k \prod_{j=1}^{m}\sum_{\ell=1}^{L} q_{\ell} f(x_{ij};\gamma_{k\ell})},$$

$$k^* = 1,\ldots,K-1,$$

$$\begin{aligned}\frac{\partial \log L(\mathbf{x}_{i*})}{\partial q_{\ell^*}} &= \sum_{k=1}^{K}\Bigg(\frac{p_k \prod_{j=1}^{m}\sum_{\ell=1}^{L} q_{\ell} f(x_{ij};\gamma_{k\ell})}{\sum_{k=1}^{K} p_k \prod_{j=1}^{m}\sum_{\ell=1}^{L} q_{\ell} f(x_{ij};\gamma_{k\ell})} \\ &\quad \times \Bigg[\sum_{j=1}^{m} \frac{f(x_{ij};\gamma_{k\ell^*}) - f(x_{ij};\gamma_{kL})}{\sum_{\ell=1}^{L} q_{\ell} f(x_{ij};\gamma_{k\ell})}\Bigg]\Bigg) \qquad \ell^* = 1,\ldots,L-1,\end{aligned}$$

$$\frac{\partial \log L(\mathbf{x}_{i*})}{\partial \mu_{k^*\ell^*}} = \frac{p_{k^*}\left[\prod_{j=1}^{m}\sum_{\ell=1}^{L} q_\ell f(x_{ij};\gamma_{k^*\ell})\right]\left[\sum_{j=1}^{m}\left\{\frac{q_{\ell^*}\frac{\partial}{\partial \mu_{k^*\ell^*}} f(x_{ij};\gamma_{k^*\ell^*})}{\sum_{\ell=1}^{L} q_\ell f(x_{ij};\gamma_{k^*\ell})}\right\}\right]}{\sum_{k=1}^{K} p_k \prod_{j=1}^{m}\sum_{\ell=1}^{L} q_\ell f(x_{ij};\gamma_{k\ell})},$$

$$k^* = 1,\ldots,K, \quad \ell^* = 1,\ldots,L,$$

$$\frac{\partial \log L(\mathbf{x}_{i*})}{\partial \sigma_{k^*\ell^*}} = \frac{p_{k^*}\left[\prod_{j=1}^{m}\sum_{\ell=1}^{L} q_\ell f(x_{ij};\gamma_{k^*\ell})\right]\left[\sum_{j=1}^{m}\left\{\frac{q_{\ell^*}\frac{\partial}{\partial \sigma_{k^*\ell^*}} f(x_{ij};\gamma_{k^*\ell^*})}{\sum_{\ell=1}^{L} q_\ell f(x_{ij};\gamma_{k^*\ell})}\right\}\right]}{\sum_{k=1}^{K} p_k \prod_{j=1}^{m}\sum_{\ell=1}^{L} q_\ell f(x_{ij};\gamma_{k\ell})},$$

$$k^* = 1,\ldots,K, \quad \ell^* = 1,\ldots,L,$$

and the partial derivatives in $\nabla_\Theta \log L(\mathbf{x}_{*j})$ are

$$\frac{\partial \log L(\mathbf{x}_{*j})}{\partial p_{k^*}} = \sum_{\ell=1}^{L}\Bigg(\frac{q_\ell \prod_{i=1}^{n}\sum_{k=1}^{K} p_k f(x_{ij};\gamma_{k\ell})}{\sum_{\ell=1}^{L} q_\ell \prod_{i=1}^{n}\sum_{k=1}^{K} p_k f(x_{ij};\gamma_{k\ell})} \times\Bigg[\sum_{i=1}^{n}\frac{f(x_{ij};\gamma_{k^*\ell}) - f(x_{ij};\gamma_{K\ell})}{\sum_{k=1}^{K} p_k f(x_{ij};\gamma_{k\ell})}\Bigg]\Bigg) \qquad k^* = 1,\ldots,K-1,$$

$$\frac{\partial \log L(\mathbf{x}_{*j})}{\partial q_{\ell^*}} = \frac{\prod_{i=1}^{n}\sum_{k=1}^{K} p_k f(x_{ij};\gamma_{k\ell^*}) - \prod_{i=1}^{n}\sum_{k=1}^{K} p_k f(x_{ij};\gamma_{kL})}{\sum_{\ell=1}^{L} q_\ell \prod_{i=1}^{n}\sum_{k=1}^{K} p_k f(x_{ij};\gamma_{k\ell})}, \qquad \ell^* = 1,\ldots,L-1,$$

$$\frac{\partial \log L(\mathbf{x}_{*j})}{\partial \mu_{k^*\ell^*}} = \frac{q_{\ell^*}\left[\prod_{i=1}^{n}\sum_{k=1}^{K} p_k f(x_{ij};\gamma_{k\ell^*})\right]\left[\sum_{i=1}^{n}\left\{\frac{p_{k^*}\frac{\partial}{\partial \mu_{k^*\ell^*}} f(x_{ij};\gamma_{k^*\ell^*})}{\sum_{k=1}^{K} p_k f(x_{ij};\gamma_{k\ell^*})}\right\}\right]}{\sum_{\ell=1}^{L} q_\ell \prod_{i=1}^{n}\sum_{k=1}^{K} p_k f(x_{ij};\gamma_{k\ell})},$$

$$k^* = 1,\ldots,K, \quad \ell^* = 1,\ldots,L,$$

$$\frac{\partial \log L(\mathbf{x}_{*j})}{\partial \sigma_{k^*\ell^*}} = \frac{q_{\ell^*}\left[\prod_{i=1}^{n}\sum_{k=1}^{K} p_k f(x_{ij};\gamma_{k\ell^*})\right]\left[\sum_{i=1}^{n}\left\{\frac{p_{k^*}\frac{\partial}{\partial \sigma_{k^*\ell^*}} f(x_{ij};\gamma_{k^*\ell^*})}{\sum_{k=1}^{K} p_k f(x_{ij};\gamma_{k\ell^*})}\right\}\right]}{\sum_{\ell=1}^{L} q_\ell \prod_{i=1}^{n}\sum_{k=1}^{K} p_k f(x_{ij};\gamma_{k\ell})},$$

$$k^* = 1,\ldots,K, \quad \ell^* = 1,\ldots,L,$$

Theoretical Properties

In this subsection, we assume the true orders of the underlying density, K and L, are known. We first introduce the result of row clustering. Define the row assignment function

$$\tau_{\text{Row}}(\mathbf{x}_{i*}) = \arg\max_k \Pr(z_i = k \mid \mathbf{x}_{i*}, \Theta).$$

For fixed $\mathbf{q}$ and row component k, we define the density function of the first layer's mixture in row composite likelihood

$$h_{\text{Row}}(x;\gamma_{k*}) = \sum_{\ell=1}^{L} q_\ell f(x;\gamma_{k\ell}),$$

where $\gamma_{k*} = (\gamma_{k1},\ldots,\gamma_{kL})$, and let its corresponding distribution be $P_{\gamma_{k*}}$. Then, given the true value of the latent row variable $z_i = k_0$, the observations within ith row $x_{i1},\ldots,x_{im}$ are i.i.d. with probability density $h_{\text{Row}}(x;\gamma_{k_0*})$.

For the consistency of the row label assignment function, we have the following conditions:

(C1) The row component k is identifiable with respect to distribution $P_{\gamma_{k*}}$, that is $P_{\gamma_{k_1*}} = P_{\gamma_{k_2*}}$ implies $k_1 = k_2$.

(C2) The set $A = \{x : h_{\text{Row}}(x;\gamma_{k*}) > 0\}$ is independent of γ_{k*}.

Considering z_i as a parameter, (C1) guarantees the identifiability of $P_{\gamma_{z_i*}}$ with respect to

z_i. (C2) guarantees the homogeneity of $P_{\gamma_{z_i *}}$ with respect to $\gamma_{z_i *}$. Now we have:

Theorem 4.1 *Suppose that the conditions (C1) and (C2) hold. Then, the proposed row label assignment function of row* i, $\tau_{Row}(\mathbf{x}_{i*})$, *converges to the true row label* $z_i = k_0$ *with probability* 1, *as the number of columns* m *goes to infinity.*

Proof. The proposed row label estimation of row i,

$$\begin{aligned}
\tau_{\text{Row}}(\mathbf{x}_{i*}) &= \arg\max_k \Pr(z_i = k \mid \mathbf{x}_{i*}, \Theta) \\
&= \arg\max_k \left\{ \frac{p_k \prod_{j=1}^{m} \sum_{\ell=1}^{L} q_\ell f(x_{ij}; \gamma_{k\ell})}{\sum_{k=1}^{K} p_k \prod_{j=1}^{m} \sum_{\ell=1}^{L} q_\ell f(x_{ij}; \gamma_{k\ell})} \right\} \\
&= \arg\max_k \left\{ p_k \prod_{j=1}^{m} \sum_{\ell=1}^{L} q_\ell f(x_{ij}; \gamma_{k\ell}) \right\} \\
&= \arg\max_k \left\{ p_k \prod_{j=1}^{m} h_{\text{Row}}(x_{ij}; \gamma_{k*}) \right\} \\
&= \arg\max_k \left\{ \frac{1}{m} \left[\log\, p_k + \sum_{j=1}^{m} \log\, h_{\text{Row}}(x_{ij}; \gamma_{k*}) \right] \right\}.
\end{aligned}$$

Since $x_{i1} \mid z_i, \ldots, x_{im} \mid z_i$ are i.i.d. with probability density $h_{\text{Row}}(x; \gamma_{z_i *})$, using Theorem 3.2 in Lehmann and Casella (1998) under (C1) and (C2),

$$P_{\gamma_{k_0 *}} \left\{ L(\gamma_{k_0 *} \mid \mathbf{x}_{i*}) - L(\gamma_{k*} \mid \mathbf{x}_{i*}) > 0 \right\} \to 1 \quad \text{as}\, m \to \infty$$

for any fixed $k \neq k_0$, where

$$L(\gamma_{k*} \mid \mathbf{x}_{i*}) = \frac{1}{m} \sum_{j=1}^{m} \log\, h_{\text{Row}}(x_{ij}; \gamma_{k*}).$$

This is equivalent to,

$$P_{\gamma_{k_0 *}} \left(\tau_{\text{Row}}(\mathbf{x}_{i*}) = z_i \right) \to 1 \quad \text{as}\, m \to \infty.$$

Similarly, the column clustering is consistent as n goes to infinity, if the similar conditions hold.

Define the column assignment function,

$$\tau_{\text{Col}}(\mathbf{x}_{*j}) = \arg\max_{\ell} \Pr(w_j = \ell \mid \mathbf{x}_{*j}, \Theta).$$

For fixed $\mathbf{p}$ and column component ℓ, we define the density function of the first layer's mixture in column composite likelihood

$$h_{\text{Col}}(x;\gamma_{*\ell}) = \sum_{k=1}^{K} p_k f(x;\gamma_{k\ell}),$$

where $\gamma_{*\ell} = (\gamma_{1\ell},\ldots,\gamma_{K\ell})$, and let its corresponding distribution be $P_{\gamma_{*\ell}}$. Then, given the true value of the latent column variable $w_j = \ell_0$, the observations within j th column $x_{1j},\ldots,x_{nj}$ are i.i.d. with probability density $h_{\text{Col}}(x;\gamma_{*\ell_0})$. We have the following conditions:

(C3) The column component ℓ is identifiable with respect to distribution $P_{\gamma_{*\ell}}$, i.e., $P_{\gamma_{*\ell_1}} = P_{\gamma_{*\ell_2}}$ implies $\ell_1 = \ell_2$.

(C4) The set $A = \{x : h_{\text{Col}}(x;\gamma_{*\ell}) > 0\}$ is independent of $\gamma_{*\ell}$.

Now we have the corollary:

Corollary 4.2 *Suppose that the conditions (C3)-(C4) hold. Then, the proposed column label assignment function of column* j*,* $\tau_{\text{Col}}(\mathbf{x}_{*j})$*, converges to the true column label* $w_j = \ell_0$ *with probability* 1*, as the number of rows* n *goes to infinity.*

Condition (C1) holds in most practical situations, but it will fail under some extreme cases, since it is possible that $P_{\gamma_{k_1*}} = P_{\gamma_{k_2*}}$ when $\gamma_{k_1*} \neq \gamma_{k_2*}$. The proposed assignment function is constructed under the assumption of pseudo-independence, which ignores the block structure among columns. If there exit two row clusters k_1 and k_2, and two column clusters ℓ_1 and ℓ_2 such that, $q_{\ell_1} = q_{\ell_2}$, $\gamma_{k_1\ell_1} = \gamma_{k_2\ell_2} \neq \gamma_{k_2\ell_1} = \gamma_{k_1\ell_2}$, and for all $\ell \neq \ell_1, \ell_2$ $\gamma_{k_1\ell} = \gamma_{k_2\ell}$, then $P_{\gamma_{k_1*}} = P_{\gamma_{k_2*}}$ while $\gamma_{k_1*} \neq \gamma_{k_2*}$.

Furthermore, when condition (C1) doesn't hold, the proposed clustering approach will not work properly, which is one of the drawbacks of the proposed composite likelihood model. Similar conclusions can be drawn for condition (C3). For example, when the array data follows the block mixture model with $\mathbf{p} = \mathbf{q} = (0.5, 0.5)$, and normal density components $N(\mu_{k\ell}, \sigma)$, where

$$(\mu_{k\ell}) = \begin{pmatrix} 0 & 1 \\ 1 & 0 \end{pmatrix},$$

then $P_{\gamma_{1*}} = P_{\gamma_{2*}}$ and $P_{\gamma_{*1}} = P_{\gamma_{*2}}$. Therefore, neither row label assignment function nor column label assignment function can cluster the data correctly.

References

1. Govaert,G. and Nadif,M. (2003) Clustering with block mixture models. *Pattern Recognit.*, **36**, 463-473.
2. Govaert,G. and Nadif,M. (2005) An EM algorithm for the block mixture model. *IEEE Trans. Pattern Anal. Mach. Intell.*, **27**, 643-647.
3. Govaert,G. and Nadif,M. (2008) Block clustering with bernoulli mixture models: Comparison of different approaches. *Comput. Stat. Data Anal.*, **52**, 3233-3245.
4. Kuruppumullage Don, P. *Estimation and model selection for block clustering with mixtures: A composite likelihood approach*. PhD dissertation, Pennsylvania State University.
5. Lindsay,B.G. (1988) Composite likelihood methods. *Contemp. Math.*, **80**, 221-39.
6. Lindsay,B.G., Yi,G.Y. and Sun,J. (2011) Issues and strategies in the selection of composite likelihoods. *Stat. Sinica*, **21**, 71.
7. McLachlan,G. and Peel,D. (2004) *Finite mixture models*. John Wiley & Sons, Hoboken.
8. Gao,X. and Song,P.X.K. (2010) Composite likelihood Bayesian information criteria for model selection in high-dimensional data. *JASA*, **105**, 1531-1540.

9. Lehmann,E.L. and Casella,G. (1998) *Theory of point estimation.* Springer, New York.
